# Therapeutic Strategy for Acute Spinal Cord Contusion Injury: Cell Elimination Combined with Microsurgical Intervention

**DOI:** 10.1371/journal.pone.0000565

**Published:** 2007-07-18

**Authors:** Nurit Kalderon, Manickam Muruganandham, Jason A. Koutcher, Melissa Potuzak

**Affiliations:** 1 Molecular Pharmacology & Chemistry Program, Sloan-Kettering Institute for Cancer Research, New York, New York, United States of America; 2 Department of Medical Physics, Memorial Sloan-Kettering Cancer Center, New York, New York, United States of America; 3 Department of Radiology, Memorial Sloan-Kettering Cancer Center, New York, New York, United States of America; Emory University, United States of America

## Abstract

**Background:**

No cure is available for human spinal cord injury. Cell elimination by localized radiation therapy that is timed within 2–3 weeks postinjury can facilitate repair of structure and function in transected rat spinal cord. In pilot studies in contusion spinal cord injury, a model similar to crush/fracture injury in human, we did not observe the expected beneficial effects of radiation therapy. Long forgotten data show that in contusion/crush injury, fluid accumulation from hemorrhage is critical. Alfred Reginald Allen observed that the most devastating sequelae in contusive injury are secondary to fluid accumulation which could be alleviated by surgical intervention, midline slits (myelotomy) at the lesion site.

**Methods and Findings:**

Here, we tested whether release of fluid buildup by microsurgery (partial myelotomy) would affect the structural outcome of radiation therapy in the severely contused rat spinal cord. Surgical intervention alone significantly enhanced tissue and functional preservation in the contused cord, thus confirming Allen's observations. Combining partial myelotomy with radiation therapy that is specifically timed postinjury elicited substantial beneficial therapeutic outcome; it led to significant increase in tissue repair/preservation compared with the group that received surgical intervention only, as determined by histology and in vivo MRI. Altogether, the combined treatments led to a 1.8 fold increase in tissue repair/preservation as compared with the contused group.

**Conclusions:**

The data suggest that a clinical protocol could be developed to treat acute human spinal cord injury through conventional clinical procedures, a combination of microsurgical manipulation and radiation therapy. These also suggest it is imperative to first prevent the secondary damage caused by fluid accumulation for a cure to be possible.

## Introduction

The pathologic hallmark of spinal cord injury is progressive tissue decay at the damage site resulting in enlarging cavitation instead of wound healing, as was observed both in human [1]–[2] and animal models [3]–[5]. Longitudinal studies suggest that the onset of tissue decay does not occur immediately but rather late in the second week and/or during the third week postinjury [4], [6]; and, that at first normal wound healing processes take place (see figure 2 in Kalderon and Fuks [4]). Similar observations about the delayed onset of tissue decay can be deduced from several studies in contusion injury [7]–[9].

It was shown, in transection injury, that the pathologic outcome can be manipulated and wound repair obtained by using, within a critical time window, a non-invasive clinical procedure to eliminate some cells generated at the lesion site [4]–[5]. Specifically, localized radiation therapy ―as used clinically for treating cancer― given at the lesion site in transected rat spinal cord within 2–3 weeks postinjury can for the most part halt the onset and progression of decay processes; consequently, some structural and functional repair is facilitated [4]–[5], [10].

The objective is to develop this strategy of cell-elimination as a therapy for contusion injury that is most similar to crush and fracture spinal cord injuries in the human [11]–[13]. In a pilot study, we treated the contusion site with a radiation therapy protocol that led to repair in the transection model. However, we could not detect any beneficial effects on wound repair. Similar results, lack of any beneficial effects in the contusion injury model, were observed by Zeman et al. [14] when using a single-dose irradiation protocol as used previously by Kalderon and Fuks [4] in transection injury. The striking difference between transection *vs.* contusion injury in the effectiveness of radiation therapy to facilitate repair suggested to us that the reason may lie in the physical consequences following these two types of injuries possibly related to fluid buildup [11], [15]. In transection any fluid can be easily released because the dura and the cord are cut open; whereas, in contusion the fluid remains enclosed and is accumulating within the damaged tissue.

Alfred R. Allen, about a century ago, developed the weight-drop device to produce impact injury in an animal model that is similar to crush injuries due to fracture dislocation of the spinal column in human [11]. Using his device, Allen hypothesized that there are two stages in impact injury, the immediate direct injury to the fiber tracts and the long-term secondary damage caused by the hemorrhage and fluid buildup due to injured blood vessels [11]. Accordingly, Allen asked: “What effect would a median longitudinal incision into the spinal cord have on subjects submitted to hyperimpact?” Allen's data suggested that medial longitudinal incision (myelotomy) within a few hours postinjury was beneficial both structurally and functionally in human [15] and in dog [11], [15]. Allen's observations were confirmed 42 years later in an animal model [16].

Here, we tested whether release of fluid build-up by midline incision would affect the structural outcome of radiation therapy in the contused rat spinal cord. Since the dura in rat is very thin, it is impossible to seal it off after cutting it open, as done in myelotomy; therefore, we used a modified, partial myelotomy which does not require any suturing of the dura. We first tested this hypothesis in a moderate contusion as defined by Basso et al. [3]. Since the preliminary data were very exciting we switched to the most clinically relevant injury, to severe contusion as defined by Basso et al. [3], in which the spared structure and motor function are minimal. The data reported here are from the severe contusion injury model.

## Materials and Methods

### Surgical procedures

Adult Sprague-Dawley female rats (Charles River Breeding Laboratories), 3–6 mos old were used. Laminectomy was performed at T10 under anesthesia [chloral hydrate, 0.042 g per 100 g body weight by i.p. injection, and Torbugesic (butorphanol tartarate), 0.02 mg per 100 g body weight by s.c. injection] as described previously [4]–[5]. Contusion injury was performed with a New York University (NYU) weight-drop device [12], dropping the 10-g rod (2.5 mm in diameter) on the exposed spinal cord surface. Moderate or severe contusion injuries were performed by dropping the weight from 12.5 mm or 50 mm, respectively [3]. Since the dura in rat is very thin, it is impossible to seal it off after cutting it open, as done in myelotomy; thus, we used a modified, partial myelotomy which did not require suturing of the dura and which is very similar to the procedure used for grafting cells into the lesion site, e.g., McDonald et al. [17]. The modified midline incision, which is also termed here microsurgical manipulation/intervention, was performed by a perpendicular stabbing at 5 points along the midline of the lesion site with a 26G needle through the dura to the bottom of the cord. No special care was needed apart from bladder expression, which were emptied manually 3–4 times a day within the 1st wk postinjury and 1–2 times per day thereafter. The animal care was in accordance with the Institutional Animal Care and Use Committee guidelines.

### Radiation therapy

Irradiation was delivered by an x-ray generator, a hybrid orthovoltage unit operating at 250 kVp, 10 mA with 0.25 mm Cu filtration, at a dose rate 101.7 cGy/min, at a distance of 50 cm from the skin as previously described [5]. Briefly, treatment was delivered through a posterior approach, centered at the site of lesion and exposure field measured 25 mm × 20 mm (long × wide). The radiation protocol consisted of 10 fractions of 2 Gy per day which were delivered daily for 7 consecutive days, resting 3 days and then resuming delivery for 3 more consecutive days, starting on day 10 postinjury with a total dose of 20 Gy. Selection for irradiation was random.

### MRI in vivo

Magnetic resonance imaging (MRI) scans of the lesion site of the spinal cord of anesthetized rat [5] were obtained on a 4.7T/33 cm bore CSI Omega imaging spectrometer (Bruker, Fremont, CA) equipped with shielded gradients (70 mT/m) and a 3 cm square surface coil. The rat was placed in a circular plastic holder (half a tube, 7.2 cm diameter) lying flat on its belly and the surface coil was centered dorsally at the lesion site. T_2_–weighted axial (transversal) images of the cord were acquired using a spin-echo pulse sequence with the following parameters: TR (repetition time)  =  3500 ms; TE (echo time)  =  40 ms, 4 excitations, 128 × 128 imaging matrix, at FOV (field of view)  =  30 mm, 1.5 mm thick slice, collecting 8 adjacent slices with 0.5 mm gap. Image processing was performed on a Unix workstation using Interactive Data Language software (Research System, Inc., Boulder, Co). For data analysis, the images were further converted into TIFF files.

### Treadmill exercising

Treadmill exercising to improve the spared/restored hindlimb function was performed according to the protocol developed for spinalized cats [18], using a rat treadmill (AccuScan Instruments, Inc.). Briefly, a special stationary plastic platform was manufactured and placed above the moving belt (1 cm above the belt). For the training, the rats were put in the treadmill and their forelimbs placed on the stationary platform while their hindlimbs were placed on the belt; the hindquarters were suspended above the surface with a strap held around the belly. To initiate hindlimb movement the tail was occasionally lightly pinched [18]. Each rat was individually exercised; exercise was given 5 days per wk, 10 min per day starting at 7–11 days postinjury.

### Evaluation of motor function

Locomotor behavior was monitored and scored according to the Basso, Beattie and Bresnahan (BBB) open field test [19]. Locomotor behavior was tested in the first 2 weeks postinjury once every 2–4 days and later once a week until the end of the experiments, 3 months postinjury. Testing was blinded.

### Tissue harvest and histology

Harvest of cord tissue samples containing the lesion site and histology were performed as previously described [4]. Frozen and cryo-protected cord samples, at least 15 mm long with the lesion site at the center, were cryostat-sectioned (15 µm thick) in a sagittal plane; the serially collected sections were stained for routine histology with thionin and examined by light microscope.

### Quantitative morphometry

Quantitative analysis for degree of tissue repair/preservation was performed by morphometry of the acquired *in vivo* MRI and *ex vivo* light microscope digital images of the lesion site with Image-Pro® Plus 4 software (Media Cybernetics, Silver Spring, MD). The entire process was blinded.

#### MRI morphometry

Eight serial axial images were collected from the lesion site of each of the cords; these were taken from the same cord level, i.e., the lesion epicenter was located at the 3rd and/or 4th slice whereas the 1st slice was mostly intact. The cord area in each of these eight axial slices was measured. To normalize the measurements, the area measured in each of the slices, 2nd through 8th, was divided by the area measured in the 1st slice.

#### Ex vivo morphometry

The major consideration for selecting the appropriate procedure of analysis was the size and shape of the lesion. If one wants to see effects of tissue repair, to prevent any bias in the data analysis, one should avoid any data collection from the boundary region between the direct impact site and the surrounding unimpacted areas. The NYU impactor used here is a cylinder with a surface diameter of 25 mm, which was dropped on the center of the dorsal part of the cord which is about 4 mm wide. The surface area of the lesion is 4.9 mm^2^. Therefore, to prevent any bias and inclusion of false repair, the quantitative analysis was performed only on the very central portion (sagittal view) of the cord that is about 1 mm wide and which includes about 50–60% of the lesion site. By doing so we left no room for error: if the tissue looks better at this analyzed region we can conclude (100% confidence) that it is solely due to the treatments. Histological images were acquired with a high performance digital Retiga camera (Qimaging, Canada). The pertinent cord sagittal sections (as explained below) were digitally photographed when the lesion site was at the center of the field of view (field of view, 82 mm long). For morphometry, the epicenter of the lesion site in each of the cords was quantitatively analyzed by measuring the area of the spared tissue in a total of 6 sequential sections that were about 0.16 mm apart from each other and which were collected from the central portion of the cord representing about 50–60% of the lesion site. In measuring: spared tissue is the area of normal and non-decaying tissue and general tissue decay is identified by empty spaces and/or by clusters of debris of dead cells within the tissue.

### Statistical analysis

Morphometric data were analyzed using either the *t*-test or the one-way ANOVA with Tukey's HSD for post hoc comparisons. Locomotor behavior data were analyzed using the non-parametric, Mann-Whitney test. Significance levels were considered at *p* < 0.05. All statistical analyses were performed using Systat for Windows, version 11 (Systat Inc).

## Results

### Deleterious fate in contusion injury due to fluid buildup

**Figure 1:**
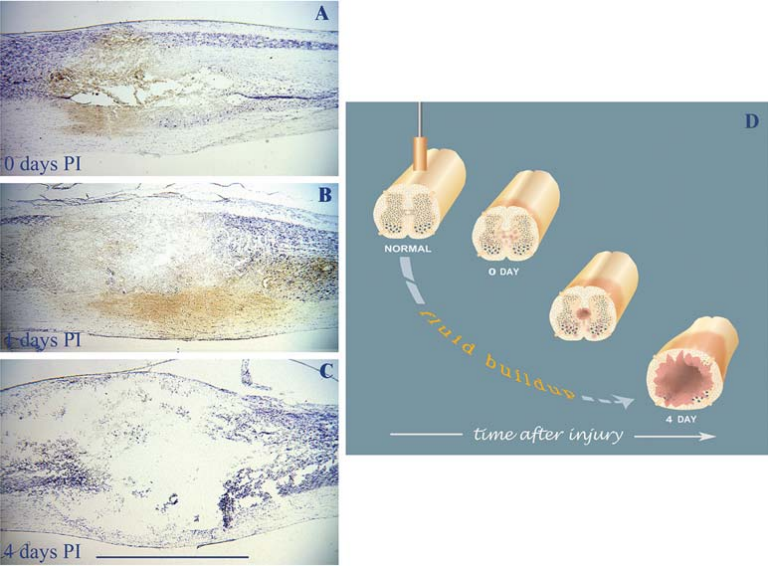
Acute fluid buildup and its detrimental outcome at the contusion lesion site. *A-C.* Longitudinal *ex vivo* view of the lesion site following moderate contusion injury, seen in thionin stained sagittal cord sections at 0, 1, and 4 days PI (postinjury), respectively (n = 3 per time-point). Note the edema, swelling and tissue rupture at 4 days PI. *D.* Schematic cartoon of fluid accumulation at the lesion site and its sequelae. Bar  =  2 mm.

**Figure 2:**
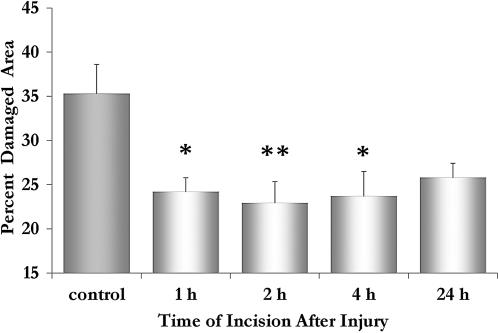
Midline incision is critical in mitigating the outcome in severe contusion injury. Plotted are the percent of damaged area in 5 severely contused cord-groups: control, and with midline slits at either 1, 2, 4, or 24 h postinjury (n = 8 per group). Note, myelotomy at 3 time points, 1-4 h, significantly reduced the lesion size and altered the outcome of injury: * *p* < 0.05; ** *p* < 0.01. Error bars, SEM.

Are the temporal events following contusion injury similar or different from those seen in transection injury? To answer, we conducted a longitudinal histological study examining consequences of a moderate contusion at different time points postinjury. Data show that the early events following contusion (Fig. 1), during the period 0–4 days postinjury, substantially differ from those observed in transection injury in that swelling, fluid buildup and tissue rupture predominate within the lesion site by day 4 postinjury (Fig. 1*C,D*) (compare with figure 2 in Kalderon and Fuks [4]). In the first day postinjury (Fig. 1*A*) the extent of the initial damage seems to be minimal, only hemorrhage was detected at the cord center whereas the dorsal/ventral white matter remained intact (Fig. 1*A*–*B*). Fluid buildup and swelling with the consequent tissue rupture occurred mostly within the first to fourth day after injury (Fig. 1*C*). Similar observations about the very early events following contusion injury were made in human [1] and in rat [20]–[21].

### Midline incision improves structural outcome in contusion injury

Next, how is the pathology and wound repair in the contused cord affected if we follow Allen's experimental paradigm of midline incision to reduce the fluid pressure? We first performed studies in a moderate contusion examining the effects of midline incision on tissue preservation/repair. The data indicated that midline incision was significantly beneficial. It enabled repair by radiation therapy. Given these data we decided to perform our study in the severe contusion injury model; studies reported below were performed in severe contusion as defined by Basso et al. [3].

We examined the role of the timing of the microsurgical intervention for reducing fluid accumulation in eliciting beneficial effects. Altogether, data show that reducing fluid accumulation by microsurgical intervention within the first 24 hours postinjury is beneficial and significantly changes the outcome of injury (Fig. 2). A severe contusion was performed (n = 40) and the injured animals were divided into 5 groups: a control group, without any added treatment, and 4 groups which underwent a midline incision at either 1, 2, 4, or 24 h postinjury. All 5 groups were processed 7 days postinjury for routine histology and the lesion sites were analyzed by quantitative morphometry for the degree of tissue damage at the lesion epicenter (Fig. 2). These data indicate that midline slits given within 1–24 h postinjury had significantly (*p*  =  0.007) altered the course of pathology at the lesion site, in that the damaged area at the lesion epicenter was reduced from 35.3% in the control group to 24.2% or 25.8% in the groups with myelotomy at 1 h or 24 h, respectively (Fig. 2). Tukey's post hoc test of pair comparison of each of the treated groups with the control group shows that surgical intervention within 1–4 h significantly (*p*≤0.03) reduced the damaged area while incision by 24 h was effective to a lesser degree (*p*  =  0.10). In comparing the 4 time points tested, no significant differences in the effectiveness of surgical intervention in reducing the damaged area could be detected between these 4 time points.

### Myelotomy is critical for repair by radiation therapy

To determine whether the fate can be mitigated and repair can be made possible in contusion injury by reducing fluid accumulation, severe contusion was performed (n = 30) and the injured animals were randomly assigned to one of the 3 conditions: no further treatment (control), midline incision (unirradiated), and midline incision followed by radiation therapy (irradiated). Midline incision was performed 1 h after the injury and radiation therapy was given at 20 Gy/10 fractions starting on day 10 postinjury. The lesion site in these 3 groups was analyzed *in vivo* at about day 50 postinjury by MRI, and *ex vivo* by histology after sacrifice at 90 days postinjury. Altogether, the data show that by preventing fluid accumulation the fate is mitigated and repair by radiation therapy is made possible. The combination of surgical intervention with radiation therapy yielded a 1.8 fold increase in tissue preservation/repair in the severely contused spinal cord.

**Figure 3:**
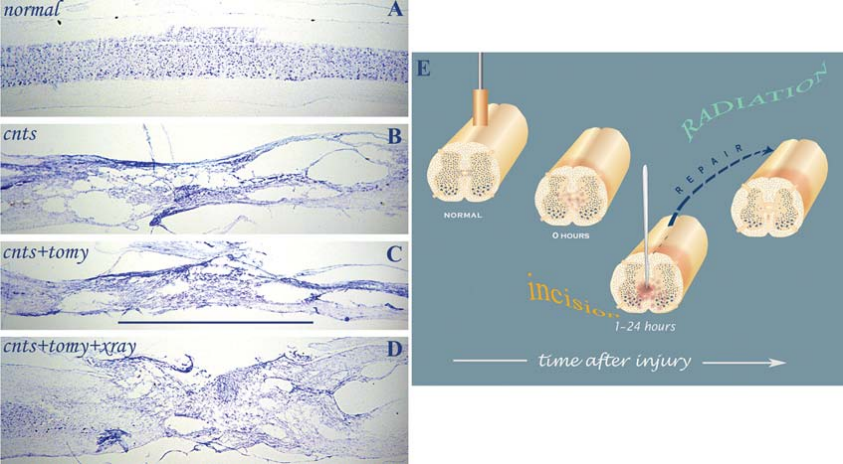
Structural repair by radiation therapy made possible by myelotomy. Micrographs of thionin-stained sagittal sections through normal intact cord and through the epicenter of 3 differently treated severely contused cords seen 90 days postinjury: *A*. normal; *B*. control, CNTS; *C*. incision, CNTS+TOMY; and *D*. incision followed by radiation therapy, CNTS+TOMY+XRAY. *E.* Schematic cartoon of the critical role of midline slits in making repair possible (compare with figure 1*D*); once the secondary damage is halted intrinsic repair can be facilitated by radiation therapy. Note, the tissue preservation and the substantial wound repair in the irradiated cord which contains in its epicenter motoneurons and other neuronal cells. Abbreviations: contusion  =  CNTS; myelotomy  =  TOMY; and radiation therapy  =  XRAY. Bar  =  3 mm.

**Figure 4:**
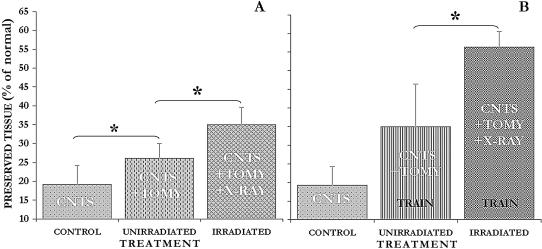
Midline incision is critical for facilitating repair in severe contusion injury: *Ex vivo* quantitative data. Degree of tissue preservation/repair, as measured by area of remaining tissue, in 3 differently treated severely contused cords: control, CNTS (n = 6); incision, CNTS+TOMY (unirradiated, n = 10); and incision followed by radiation therapy, CNTS+TOMY+XRAY (irradiated, n = 7). These 3 groups were either *A.* without training or *B*. with treadmill exercised. Note, tissue preservation was enhanced by midline incision from 19% to 26.1%, and radiation therapy increased it to 35% of the normal tissue; further treadmill exercising increased it in the unirradiated from 26.1% to 35% and in the irradiated from 35% to 56.3%. Abbreviations: contusion  =  CNTS; myelotomy  =  TOMY; and radiation therapy  =  XRAY. Significance, * *p* < 0.05. Error bars, SD.

Histologic analysis of the lesion site in the three groups (n = 23) reveals significant differences between the groups (Figs. 3–4). First, radiation therapy following midline incision prevents tissue decay and leads to wound healing and repair (Fig. 3*D*); and in some of the irradiated contused cords substantial structural repair and continuity was observed. Quantitative morphometry of the lesion site showed significant tissue preservation and/or prevention of tissue decay in the unirradiated and in the irradiated group as compared with the control group which did not undergo any added surgical manipulation (Fig. 4*A*). The percent of tissue spared in the 3 groups was calculated with respect to normal intact cord (n = 3) (Fig. 3*A*). These quantitative data show that midline incision significantly (*p* = 0.037) halts secondary damage and reduces tissue decay: 26.1% of the tissue was spared in the unirradiated group which underwent myelotomy compared to only 19% in the control group (Fig. 4*A*). Furthermore, by adding radiation therapy following the midline slits tissue preservation/repair was significantly (*p* = 0.032) enhanced from 26.1% to 35%.

### Beneficial effects of myelotomy as seen *in vivo*

**Figure 5:**
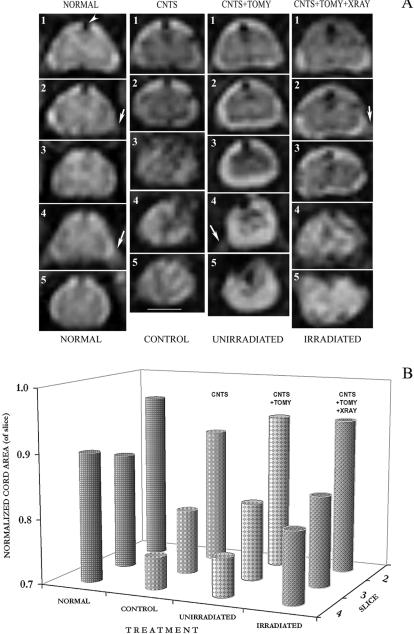
Tissue preservation/repair by myelotomy and radiation therapy *in vivo*. *A*. *In vivo* images of: five serial T_2_-weighted axial scans of normal, and of 3 differently treated severely contused cords: no added treatment, CNTS; incision, CNTS+TOMY; and incision followed by radiation therapy, CNTS+TOMY+XRAY, at 50 days postinjury (these 3 spinal cord are shown in Fig. 3 *B–D*). Each of the numbered slices was taken from the same anatomical level of the cord, e.g., slice 1 is at identical level in all 4 cords and mostly unharmed. Note, the ‘fuzziness’ of these images is an innate feature of the acquisition procedure; these are *in vivo* images that were acquired for about an hour while the animal is breathing, each showing –as a result of the field of view utilized– an enlarged cord (by x7); therefore, to obtain a better view, the figure should be held at a distance. Note, in normal cord some typical anatomical features: the butterfly shaped grey matter (in slice 1), the ventral roots (arrows, slice 2, 4), and the dorsal dark circle (arrowhead in section 1) is a blood vessel. In the injured cords, note sections 3-4 (the lesion epicenter) are abnormal and reduced in size, also the irradiated cord appears to be the least abnormal. Bar  =  2 mm. *B*. Quantitative tissue preservation by myelotomy and radiation therapy. Plotted are the normalized tissue areas in each of the serial MRI sections (2–4) of 4 groups as illustrated in panel *A* above: normal (n = 3), control (n = 6), unirradiated (n = 8), and irradiated (n = 8). Note, in the control cords tissue decay extends throughout slices 2-4 whereas in those that underwent myelotomy the damage was contained to slices 3-4. Also, tissue preservation in each of the sections is the highest in the irradiated cords in comparison with the control group. Abbreviations: contusion  =  CNTS; myelotomy  =  TOMY; and radiation therapy  =  XRAY. Note, error bars cannot be shown in a 3D plot.

*In vivo* MRI is currently the most powerful clinical tool available to detect pathologic events *in vivo* (e.g., Corbetta et al. [2]). Even though MRI is far less sensitive than histology, the beneficial effects of the microsurgery could be monitored also by *in vivo* MRI, confirming the histologic data. Quantitative morphometry of the lesion site *in vivo* (n = 22) in the 3 different groups, control, unirradiated and irradiated, shows that midline incision had beneficial effects on the course of events at the contusion site (Fig. 5). These beneficial effects can be qualitatively observed in the MRI scans by comparing the anatomy of the injured cords with that of the normal cord (Fig. 5*A*). In the injured cords, sections 3–4 (the lesion epicenter) are abnormal, reduced in size and show tissue decay/cavitation. In contrast, the irradiated cord appears to be the least abnormal and/or least reduced in size (Fig. 5*A*). The quantitative data show that the secondary damage is reduced by midline incision, and when combined with radiation therapy, tissue repair was obtained (Fig. 5*B*) in that the size of the irradiated spinal cord sections is similar to that of the normal spinal cord. Structural preservation at the lesion epicenter was significantly (*p* < 0.05) increased in the irradiated group in comparison with the control contused group, without any added surgical manipulation (Fig. 5*B*). In comparing tissue preservation between the three different groups, there is an apparent graded trend in which the irradiated and myelotomized group is the best followed by the unirradiated and myelotomized group, and the least is the control (unirradiated and unmyelotomized) group; but these differences are not statistically significant (Fig. 5*B*).

### Enhancing locomotor function by microsurgical intervention and treadmill training

**Figure 6:**
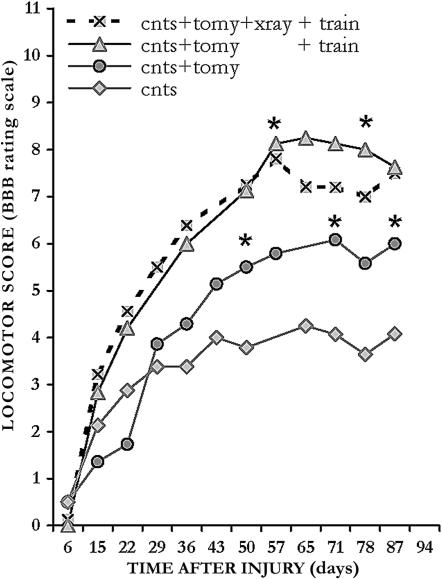
Hindlimb locomotor function improved by myelotomy and exercising. The BBB locomotor scores of the 4 differently treated severely contused groups: -⧫- no further treatment (cnts), -•- midline incision (cnts+tomy), -▴- incision and exercise (cnts+tomy+train), and -⊠- incision followed by radiation therapy and exercise (cnts+tomy+xray+train) are plotted as a function of time postinjury. Midline incision and treadmill exercising both increased the hindlimb locomotor performance: going from a severe paralysis of the hindlimb joints to a complete control of the hindlimb movement as defined by Basso et al. [19]; at about 2 months postinjury enhancement became significant and remained thereafter as such for some of the time points tested. Note, comparison for significance was performed between the following: cnts+tomy vs. cnts group; and cnts+tomy+train vs. cnts+tomy group. Significance, * *p* < 0.05. Abbreviations: contusion  =  cnts; myelotomy  =  tomy; and radiation therapy  =  xray.

The beneficial effects of midline incision were also detected in the locomotor behavior of the 3 groups as determined according to the BBB open field test (Fig. 6). Hindlimb locomotion in the unirradiated group which underwent myelotomy was significantly higher (*p* = 0.03), i.e., 6 *vs.* 4, than that of the control group. In functional terms ―according to the BBB scale― the motor improvement due to myelotomy was from a severe to mild paralysis of the hindlimb joints [19]. However, no difference was noticed in locomotion behavior between the irradiated and the unirradiated group (data not shown).

Because in severe contusion the hindlimbs are practically paralysed and have no ability to generate weight supporting stepping, for physiotherapy purposes, mostly to enhance hindlimb muscle strength, we added a treadmill exercise protocol to the unirradiated and the irradiated groups. Treadmill exercise, according to Lovely et al. [18], was started at about 7–11 days after injury and was given daily, for 10 minutes. Altogether, midline slits and exercise led to substantial functional improvement (Fig. 6).

In both groups, exercise significantly increased the BBB scoring as compared with the unexercised group (Fig. 6), i.e., 8 *vs.* 6. In functional terms ―according to the BBB scale― the motor improvement due to exercise was from a mild paralysis to a complete control of the hindlimb movement, showing sweeping or plantar placement of the paw with no weight support [19]. However, due to the insensitivity of the BBB test, no difference could be detected between the two groups that were exercised (Fig. 6). It should be noted that in a separate subsequent study when the testing procedures for recovery of motor control were kinematic [22], some substantial differences due to training were observed between irradiated and unirradiated groups in their motor performance (Ichiyama R, Potuzak M, Balak M, Kalderon N, Edgerton VR, unpublished observations).

### Treadmill training enhances tissue preservation

Most interestingly and unexpectedly, the treadmill exercise significantly affected tissue preservation. Morphometric analysis of the lesion site for tissue preservation showed a significant difference between the trained (n = 8) (Fig. 4*B*) and untrained (n = 9) (Fig. 4*A*) groups. The best preservation/repair of 56.3% was observed in the group that was irradiated and trained. Addition of exercise significantly enhanced tissue preservation in the unirradiated group from 26.1% to 35% (*p* = 0.026) and in the irradiated group from 35% to 56.3% (*p* = 0.003). Altogether, the combination of surgical intervention with radiation therapy and exercise yielded about a 3-fold increase in tissue preservation/repair in the severely contused spinal cord (Fig. 4).

## Discussion

The results of this study demonstrate two major points. First, the outcome and the secondary damage in contusive spinal cord injury can be mitigated by reducing/preventing the acute fluid accumulation at lesion site by a microsurgical intervention. Second, radiation therapy effective in repair in transection injury can also lead to significant repair in the severely contused spinal cord, provided that fluid buildup is stopped/reduced by midline slits. Further, this study also demonstrates that the degree of preservation/repair can be improved by daily exercise. These results suggest that by using simple and well-defined clinical procedures, a partial incomplete myelotomy and radiation therapy, it would be possible to develop clinical protocols for repair in human spinal cord injury. These results also suggest that to obtain optimal recovery of function it would be necessary to add functional training/exercising protocols.

Our longitudinal data about the early events following trauma indicate, as suggested by Allen [11], that fluid accumulation at the lesion site has detrimental consequences on the outcome of contusion injury. This study also confirms the ground-breaking observations made by Allen that a median longitudinal incision into the contused spinal cord has a beneficial therapeutic effect on the final outcome of the injury in that it restrains and/or prevents the propagation of the secondary damage caused by the initial trauma to the blood vessels. Finally, our data suggest that to obtain repair of the severed structure/function it is imperative first to suppress/prevent the fluid accumulation.

Unfortunately very little attention has been given to Allen's observations about the significant therapeutic power of surgical intervention in spinal cord injury. Very few studies to explore this therapeutic strategy have been performed thus far. Forty-two years after Allen made his initial observations, Freeman and Wright [16] reproduced and confirmed in rat and dog that midline incision is beneficial in preventing complete paralysis by preserving the spared cord tissue (unharmed by the primary trauma) and its related motor function. Twenty years later it was shown in the cat that a combination of myelotomy and methylprednisolone treatment prevented paralysis in 80% of the treated group [23].

Myelotomy, longitudinal continuous midline incision has been used clinically for decades in removing spinal cord tumors, causing minimal functional deficits [24]–[25]. Nevertheless, it seems that the lack of aseptic procedures in the past and the persisting biased fear of impairing motor function by the microsurgery has held back the development of myelotomy as a treatment to prevent paralysis. It is interesting to note that recent studies of stem cell grafting into the contused spinal cord have been performing by coincidence partial myelotomy, very similar to our procedure, as the cell graft in these studies was injected into the lesion site [17], [26].

It should be noted that the radiation protocol used in this study 20 Gy/10 fractions is well below the clinical tolerance of human spinal cord. Safe radiation protocols for the treatment of spinal cord tumors have been developed, employing total doses that eradicate tumors without exceeding the normal tissue tolerance. For example, the clinical tolerance dose values for the human spinal cord are 45–50 Gy and 33 Gy when delivered in daily fractions of 2 Gy and 3 Gy, respectively [27]–[29].

As for recovery of some of the interrupted function it is assumed that the insensitivity of the BBB open field test prevented detection of any recovery/restitution of function in particular in the irradiated and exercised group. It is assumed that with the development of robotic training protocols in contusion injury and related sensitive testing, e.g., as described by de Leon et al. [30], it would be possible to accurately detect/determine the extent of restoration of function. Indeed, in a separate study when the testing procedures were sensitive (kinematic testing) some substantial differences between the irradiated and unirradiated groups in their hindlimb motor performance were observed. The trained irradiated group had the best performance (Ichiyama R, Potuzak M, Balak M, Kalderon N, Edgerton VR, unpublished observations).

Finally, the beneficial effects of exercising on cord tissue preservation and motor function sparing are most intriguing. At this stage, the mechanism for these beneficial effects is unclear. Presumably, prevention of atrophy of the spared motoneurons at the lesion site has an important role, e.g., [31]–[32].

No therapies are currently available for the primary damage in spinal cord injury. Only very limited therapeutic means are presently available for spinal cord injury treatment; these are aimed at reducing the degree/extent of the secondary damage during the very early acute phases following spinal cord injury. In fact, the only generally accepted acute intervention after spinal cord injury is administration within 8 hours after injury of high doses of the steroid methylprednisolone [33]. Animal studies suggest that the beneficial effect of methylprednisolone in spinal cord injury is in reducing the edema [34]–[35].

This study provides a proof of principle; it demonstrates that a therapy for the primary damage in spinal cord injury is possible through conventional clinical procedures, a combination of microsurgical manipulation and radiation therapy. Thus, the door is also open to other procedures that could be added to the clinical protocol developed in this study to further enhance the degree of structural and functional repair in spinal cord injury. It is open to the existing procedures, such as methylprednisolone [33] and/or to the newly developed, such as elevation of internal of cAMP levels [36].

## References

[1] Tator CH, Koyanagi I (1997). Vascular mechanisms in the pathophysiology of human spinal cord injury.. J Neurosurg.

[2] Corbetta M, Burton H, Sinclair RJ, Conturo TE, Akbudak E (2002). Functional reorganization and stability of somatosensory-motor cortical topography in a tetraplegic subject with late recovery.. Proc Natl Acad Sci USA.

[3] Basso DM, Beattie MS, Bresnahan JC (1996). Graded histological and locomotor outcomes after spinal cord contusion using the NYU weight-drop device versus transection.. Exp Neurol.

[4] Kalderon N, Fuks Z (1996). Structural recovery in lesioned adult mammalian spinal cord by x-irradiation of the lesion site.. Proc Natl Acad Sci USA.

[5] Kalderon N, Xu S, Koutcher JA, Fuks Z (2001). Fractionated radiation facilitates repair and functional motor recovery after spinal cord transection in rat.. Brain Res.

[6] Kalderon N (2005). Cell elimination as a strategy for repair in acute spinal cord injury.. Curr Pharm Design.

[7] Popovich PG, Horner PJ, Mullin BB, Stokes BT (1996). A quantitative spatial analysis of the blood-spinal cord barrier.. Exp Neurol.

[8] Bilgen M, Abbe R, Narayana PA (2001). Dynamic contrast-enhanced MRI of experimental spinal cord injury: *In vivo* serial studies.. Magn Reson Med.

[9] Loy DN, Crawford CH, Darnall JB, Burke DA, Onifer SM (2002). Temporal progression of angiogenesis and basal lamina deposition after contusive spinal cord injury in the adult rat.. J Comp Neurol.

[10] Kalderon N, Fuks Z (1996). Severed corticospinal axons recover electrophysiologic control of muscle activity after x-ray therapy in lesioned adult spinal cord.. Proc Natl Acad Sci USA.

[11] Allen AR (1911). Surgery of experimental lesion of spinal cord equivalent to crush injury of fracture dislocation of spinal column.. J Am Med Assoc.

[12] Gruner JA (1992). A monitored contusion model of spinal cord injury in the rat.. J Neurotrauma.

[13] Stokes BT, Jakeman LB (2002). Experimental modeling of human spinal cord injury: a model that crosses the species barrier and mimics the spectrum of human cytopathology.. Spinal Cord.

[14] Zeman RJ, Feng Y, Peng H, Visintainer PF, Moorthy CR (2001). X-irradiation of the contusion site improves locomotor and histological outcomes in spinal cord-injured rats.. Exp Neurol.

[15] Allen AR (1914). Remarks on the histopathological changes in the spinal cord due to impact. An experimental study.. J Nerv Ment Dis.

[16] Freeman LW, Wright TW (1953). Experimental observations of concussion and contusion of the spinal cord.. Ann Surg.

[17] McDonald JW, Liu X-Z, Qu Y, Liu S, Mickey SK (1999). Transplanted embryonic stem cells survive, differentiate and promote recovery in injured rat spinal cord.. Nature Med.

[18] Lovely RG, Gregor RJ, Roy RR, Edgerton VR (1986). Effects of training on the recovery of full-weight bearing stepping in adult spinal cat.. Exp Neurol.

[19] Basso DM, Beattie MS, Bresnahan JC (1995). A sensitive and reliable locomotor rating scale for open field testing in rats.. J Neurotrauma.

[20] Falconer JC, Narayana PA, Bhattacharjee MB, Liu SJ (1994). Quantitative MRI of spinal cord injury in a rat model.. Magn Reson Med.

[21] Merola A, O'Brien MF, Castro BA, Smith DAB, Eule JM (2002). Histologic characterization of acute spinal cord injury treated with intravenous methylprednisolone.. J Orthop Trauma.

[22] Timoszyk WK, De Leon RD, London N, Roy RR, Edgerton VR (2002). The rat lumbosacral spinal cord adapts to robotic loading applied during stance.. J Neurophysiol.

[23] Campbell JB, DeCrescito V, Tomasula JJ, Demopoulos HB, Flamm ES (1973). Experimental treatment of spinal cord contusion in the cat.. Surg Neurol.

[24] Markham JW, Walker AE (1951). Surgery of the spinal cord and vertebral column.. A History of Neurological Surgery..

[25] Cooper PR, Epstein F (1985). Radical resection of intramedullary spinal cord tumors in adult.. J Neurosurg.

[26] Hofstetter CP, Schwarz EJ, Hess D, Widenfalk J, El Manira A (2002). Marrow stromal cells form guiding strands in the injured spinal cord and promote recovery.. Proc Natl Acad Sci USA.

[27] Marcus RB, Million RR (1990). The incidence of myelitis after irradiation of the cervical spinal cord.. Int J Radiat Oncol Biol Phys.

[28] Schultheiss TE (1990). Spinal cord radiation “tolerance”: Doctrine versus data.. Int J Radiat Oncol Biol Phys.

[29] Khan DC, Malhotra S, Stevens RE, Steinfield AD (1999). Radiotherapy for the treatment of giant cell tumor of the spine: a report of six cases and review of the literature.. Cancer Invest.

[30] de Leon RD, Kubasak MD, Phelps PE, Timoszyk WK, Reinkensmeyer DJ (2002). Using robotics to teach the spinal cord to walk.. Brain Res Rev.

[31] Wu CW-H, Kaas JH (2000). Spinal cord atrophy and reorganization of motoneuron connections following long-standing limb loss in primates.. Neuron.

[32] Qi H-X, Phillips WS, Kaas JS (2004). Connections of neurons in the lumbar ventral horn of spinal cord are altered after long-standing limb loss in a macaque monkey.. Somatosens Mot Res.

[33] Bracken MB, Shepard MJ, Collins WF, Holford TR, Young W (1990). A randomized, controlled trial of methylprednisolone or naloxone in the treatment of acute spinal-cord injury.. N Engl J Med.

[34] Narayana P, Abbe R, Liu SJ, Johnson D (1999). Does loss of gray- and white-matter contrast in injured spinal cord signify secondary injury? In vivo longitudinal MRI study.. Magn Reson Med.

[35] Rabchevsky AG, Fugaccia I, Sullivan PG, Blades DA, Scheff SW (2002). Efficacy of methylprednisolone therapy for the injured rat spinal cord.. J Neurosci Res.

[36] Pearse DD, Pereira FC, Marcillo AE, Bates ML, Berrocal YA (2004). cAMP and Schwann cells promote axonal growth and functional recovery after spinal cord injury.. Nature Med.

